# Absence of Non-Canonical, Inhibitory *MYD88* Splice Variants in B Cell Lymphomas Correlates With Sustained NF-κB Signaling

**DOI:** 10.3389/fimmu.2021.616451

**Published:** 2021-06-07

**Authors:** Yamel Cardona Gloria, Stephan H. Bernhart, Sven Fillinger, Olaf-Oliver Wolz, Sabine Dickhöfer, Jakob Admard, Stephan Ossowski, Sven Nahnsen, Reiner Siebert, Alexander N. R. Weber

**Affiliations:** ^1^ Department of Immunology, University of Tübingen, Tübingen, Germany; ^2^ Cluster of Excellence iFIT (EXC 2180) “Image-Guided and Functionally Instructed Tumor Therapies”, University of Tübingen, Tübingen, Germany; ^3^ Interdisciplinary Center for Bioinformatics, University of Leipzig, Leipzig, Germany; ^4^ Bioinformatics Group, Department of Computer, University of Leipzig, Leipzig, Germany; ^5^ Transcriptome Bioinformatics, Leipzig Research Center for Civilization Diseases (LIFE), University of Leipzig, Leipzig, Germany; ^6^ Quantitative Biology Center (QBiC), University of Tübingen, Tübingen, Germany; ^7^ Institute of Medical Genetics and Applied Genomics, University of Tübingen, Tübingen, Germany; ^8^ Institute of Human Genetics, Ulm University and Ulm University Medical Center, Ulm, Germany; ^9^ Institute of Human Genetics, Christian-Albrechts-University, Kiel, Germany; ^10^ Deutsches Konsortium für Translationale Krebsforschung (DKTK; German Cancer Consortium), Partner Site Tübingen, Department of Immunology, University of Tübingen, Tübingen, Germany

**Keywords:** MYD88, B cell lymphoma, DLBCL - diffuse large B cell lymphoma, NF- kappa B, TLR - Toll-like receptor, alternative splicing, negative feedback loop

## Abstract

Gain-of-function mutations of the TLR adaptor and oncoprotein MyD88 drive B cell lymphomagenesis *via* sustained NF-κB activation. In myeloid cells, both short and sustained TLR activation and NF-κB activation lead to the induction of inhibitory *MYD88* splice variants that restrain prolonged NF-κB activation. We therefore sought to investigate whether such a negative feedback loop exists in B cells. Analyzing *MYD88* splice variants in normal B cells and different primary B cell malignancies, we observed that *MYD88* splice variants in transformed B cells are dominated by the canonical, strongly NF-κB-activating isoform of *MYD88* and contain at least three novel, so far uncharacterized signaling-competent splice isoforms. Sustained TLR stimulation in B cells unexpectedly reinforces splicing of NF-κB-promoting, canonical isoforms rather than the ‘MyD88s’, a negative regulatory isoform reported to be typically induced by TLRs in myeloid cells. This suggests that an essential negative feedback loop restricting TLR signaling in myeloid cells at the level of alternative splicing, is missing in B cells when they undergo proliferation, rendering B cells vulnerable to sustained NF-κB activation and eventual lymphomagenesis. Our results uncover *MYD88* alternative splicing as an unappreciated promoter of B cell lymphomagenesis and provide a rationale why oncogenic *MYD88* mutations are exclusively found in B cells.

## Highlights

- In human B cells the TLR adaptor and oncogene, *MYD88*, can give rise to at least 8 mRNA splice variants with different signaling capabilities.
- Unlike myeloid cells, transformed B cells and cells with sustained TLR/NF-κB activation show a preference for NF-κB-promoting canonical *MYD88* splice variants.
- The negative feedback loop of inducing signaling incompetent splice variants is absent in proliferating B cells and may render them susceptible to lymphomagenesis.

## Introduction

MyD88 has long been studied as an adaptor molecule for Toll-like receptor (TLR) and Interleukin-1 receptor (IL-1R) signaling in innate immunity (1). Its pivotal role is strikingly illustrated by the fact that loss-of-function mutations lead to severe immunodeficiency, whereas gain-of-function mutations promote oncogenesis: For example, rare dysfunctional alleles of *MYD88* compromise formation of the MyD88-mediated post-receptor complex (2), the so-called Myddosome (3, 4). Its assembly is a pre-requisite for effective activation of the IL-1R-associated kinases (IRAKs) 2 and 4 and eventual activation of NF-κB and mitogen activated protein (MAP) kinases (1). Patients carrying loss-of-function *MYD88* alleles consequently fail to respond to microbial TLR agonists and IL-1 and thus do not mount a sufficient innate immune response against pyogenic bacteria, leading to insufficient immunity and frequent premature death (5). Conversely, *MYD88* mutations leading to constitutive Myddosome assembly (6), most notably the mutation Leu 265 to Pro (L265P) (7), are oncogenic and associated with sustained NF-κB signaling. L265P drives lymphoproliferation in murine models (8). In humans, L265P is highly prevalent in various B cell malignancies (7) but absent in other, e.g. myeloid, hematopoietic (8) malignancies. Its strict occurrence in B cell malignancies has highlighted L265P’s diagnostic, chemo- and immunotherapeutic potential (9–11) but also posed the questions why only B cells are vulnerable to *MYD88* gain-of-function mutations? Additionally, the varying frequency of the L265P mutation in different B cell malignancies has been puzzling: Although the MyD88 L265P mutation may be found in up to 90% of Waldenström’s Macroglobulinemia patients (12), in diffuse large B cell lymphoma (DLBCL) and chronic lymphocytic leukemia (CLL) only 30 or 4% of patients carry this or other known gain-of-function *MYD88* mutations, depending on subtype (7, 13). Thus, other mechanisms apart from mutation of *MYD88* appear to operate in L265P-negative patients, whereas a consistent “NF-κB signature” has been recognized as a unifying feature for most of these B cell malignancies (14–16).

**Figure 1:**
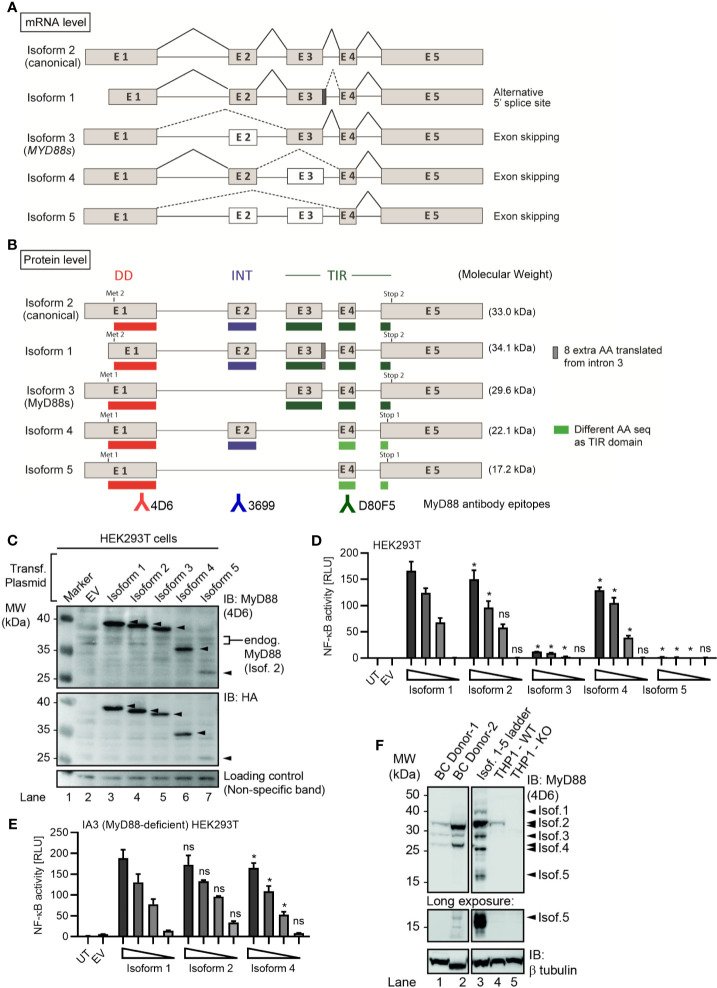
Several alternative MyD88 isoforms support NF-κB signaling**. (A, B)** Schematic representation of *MYD88* isoforms on mRNA **(A)** and protein **(B)** level according to references in **Table 1**. **(B)** Illustration of target epitopes of the different antibodies used in this study. **(C–E)** HEK293T cells were transfected with plasmids for different *MYD88* splice isoforms and lysates analyzed for expression or pathway activation by immunoblot (**C**, n=3) or NF-κB dual luciferase assay (**D**, n=4), respectively. **(E)** as in D but using MyD88-deficient I3A cells (n=3). **(F)** Immunoblot of primary B cell lysates from two different donors, lysates of HEK293T transfected with untagged isoforms 1 to 5 (‘Isof. 1-5 ladder’) and MyD88-competent or deficient (KO) THP-1 reporter cells. In C-E one representative of ‘n’ technical replicates is shown as mean + SD from three repeats. ns, non-significant; * p<0.05 according to two-way ANOVA comparing to isoform 1 **(D, E)**.

The activation of NF-κB is also a primary outcome of MyD88-dependent signaling in myeloid cells (1). However, negative feedback on NF-κB signaling by alternative splicing seemingly operates in myeloid cells: TLR stimulation with LPS leads to the upregulation of a splice variant, then termed ‘MyD88 short’ (MyD88s, here also referred to as isoform 3, see **Figures 1A, B** and **Table 1**) (17). Conversely to constitutive splicing (18), alternative splice variants arise from “alternative” splice sites in pre-mRNAs, that trigger, for example, exon skipping, alternative 5’ or 3’ splice site usage within exon or intron sequences or intron retention. The resulting transcripts may be subject to frame shifts, premature termination codons and/or non-sense mediated decay (NMD) (18, 19). Collectively, >90% of human multi-exon genes are subject to alternative splicing which greatly expands the diversity and function of the proteome (20, 21). In eukaryotes the spliceosome, where so-called splice factors (SFs) cooperate with five small nuclear ribonucleoprotein complexes (U1, U2, U4/U6, and U5), recognizes and assembles on introns to cleave and ligate RNA molecules for intron removal, generating protein-coding mRNAs (22). The spliceosome catalyzes splicing with high precision, but also displays high flexibility to regulatory signals for rapid responses, such as alternative splicing. Such a direct link between regulatory signals and innate immunity was recently proposed for the SF3A and SF3B mRNA splicing as both factors were shown to connect TLR signaling with the regulation of MyD88s (23, 24).

**Table 1:** *MYD88* splice isoforms.

MyD88 isoforms	mRNA			Protein			Expression construct MW (incl. Strep-HA tag; kDa)*
	Reference ID	CDS (bp)		Reference ID	Length (aa)	MW (kDa)	
Isoform 1	ENST00000421516.3 NM_001172567.2	915		ENSP00000391753 NP_001166038.2	304	34.1	40.5
Isoform 2	ENST00000396334.8 NM_002468.5	891		ENSP00000379625 NP_002459.3	296	33.0	37.4
Isoform 3 (MyD88s)	ENST00000417037.7 NM_001172568.2	795		ENSP00000401399 NP_001166039.2	264	29.6	34.7
Isoform 4	ENST00000651800.1 NM_001172569.3	615		ENSP00000499012 NP_001166040.2 Uniprot: Q99836-3	204	22.1	27.2
Isoform 5	ENST00000650112.1 NM_001172566.2	480		ENSP00000497991 Uniprot: Q99836-4	159	17.2	22.8
Isoform 6	ENST00000652213 NM_001365876.1	738		ENSP00000498576 NP_001352805.1	245	27.1	34.9^§^
Isoform 7	NM_001365877.1	642		NP_001352806.1	213	23.5*	29.9^§^
Isoform 8	ENST00000652590.1	723		n/a (new)	240	26.4*	32.8^§^

Reference IDs from Ensembl and NCBI. ENST: cDNA sequence, ENSP: protein sequence, NM: curated NCBI mRNA; Protein-coding transcript, NP, NCBI protein coding sequence; Strep-HA, Strep III - Hemagglutinin tag; n/a, not available. *Values were calculated using ExPASy. ^§^ Generated constructs use Met1 as start codon.

MyD88s (isoform 3) represents an alternatively spliced in-frame deletion of exon 2 and thus a MyD88 variant significantly shorter than the canonical isoform 2: Whereas isoforms 1 and 2 contain the canonical N-terminal death domain (DD), central intermediate domain (ID) for IRAK recruitment, and C-terminal Toll/IL-1R (TIR) domain for TLR binding, MyD88s (isoform 3) lacks the ID. The ID has been proposed to couple activated TLRs to the IRAK-containing Myddosome and thus transduce incoming signal (25). Hence, MyD88s is signaling-incompetent. Even though its characterization has been limited to myeloid and epithelial cells, MyD88s (isoform 3) by many has been considered a primary negative regulator of this pathway and part of an essential negative feedback loop induced upon TLR signaling in myeloid cells and epithelial (26–28). Isoform 1, the first reference sequence described, represents the longest transcript and translated protein for MyD88 by taking an alternative donor splice site 24 nt downstream of exon 3, adding 8 amino acids within the TIR domain. Apart from isoforms 1-3, two additional splice isoforms of *MYD88* have since been described, namely, isoforms 4 and 5 (**Figures 1A, B** and **Table 1**), whose properties have been less studied. Additionally, whether alternative splicing and feedback regulation is operable in other, non-myeloid immune cells has not been addressed.

We speculated that if a negative feedback loop existed in B cells, TLR activation should also induce MyD88s (isoform 3) and thereby limit ongoing signaling. Interestingly, we found here that B cells only transiently induce isoform 3 upon short exposure to TLR agonists, but extended TLR-MyD88 stimulation rather maintained the canonical isoform. Our data thus indicates that in B cells an isoform 3-mediated negative feedback loop does not seem to restrain NF-κB long-term; rather, extended TLR stimulation drives the canonical, i.e. NF-κB promoting, isoform and thus does not restrict extended NF-κB activation by diverting transcripts to less signaling-competent isoforms like MyD88s (isoform 3) as in myeloid cells. In line with this, primary B cell malignancies showed significantly higher degrees of the canonical *MYD88* splice isoform and include transcripts for an additional three hitherto unrecognized *MYD88* splice isoforms. Our data warrant a re-evaluation of previously assumed myeloid cell derived concepts of *MYD88* splicing and NF-κB regulation in human primary cells, especially B cells, and provide an explanation for the susceptibility of B cells to oncogenic *MYD88* mutation.

## Materials and Methods

### Study Participants and Sample Acquisition

All patients and healthy blood donors included in this study provided their written informed consent before study participation. Approval for use of their biomaterials was obtained by the local ethics committee at the University Hospitals of Tübingen; Germany, in accordance with the principles laid down in the Declaration of Helsinki as well as applicable laws and regulations. Patient recruitment, sample acquisition and preparation of B cell lymphoma, CLL and ovarian cancer patients are described below. Healthy blood donors were recruited at the Interfaculty Institute of Cell Biology, Department of Immunology, University of Tübingen; Germany.

### Isolation and Stimulation of Primary Human Immune Cells

Peripheral blood mononuclear cells (PBMCs) from healthy donors were isolated from whole blood or buffy coats (University Hospital Tübingen Transfusion Medicine) using Ficoll density gradient purification, primary B cells from PBMCs using B Cell Isolation Kit II (Miltenyi Biotec, >90% purity by anti-CD19 staining) and hMoMacs using Monocyte attachment Medium (PromoCell). B cells and hMoMacs were stimulated with 200 ng/ml LPS (from *E. coli* K12, Invivogen) or 2.5 µg/ml CpG 2006 (TIB MOLBIOL) for the indicated time periods. B cells were also stimulated with 2.5 µg/ml CpG 2006 and 5 µg/mL anti-human IgM (Fc5µ, Jackson Immuno Research) for proliferation assays. Carboxyfluorescein-succinimidyl ester (CFSE, Life Technologies) was used to track cell proliferation. Flow cytometry (BD FACSCanto II) was analyzed using FlowJo PC version 10. Further details in **Supplemental Information**.

### Plasmid Constructs

N-terminally StrepIII-Hemagglutinin (HA) tagged and untagged *MYD88* isoform expression constructs were based on the reference sequences listed in **Table 1** and generated by gene synthesis (Genewiz; Germany) or PCR cloning and verified by DNA sequencing. Further details in **Supplemental Information**.

### Cell Cultures

All HEK293T and DLBCL cell lines were described and cultured as previously (6). THP-1 WT and MyD88-deficient cells were a kind gift from V. Hornung, Gene Center, Munich, Germany. THP-1 WT and MyD88-KO Dual reporter cells were provided by R. Amann, University of Tübingen, Germany. Further details in **Supplemental Information**.

### Dual Luciferase Assay

Dual luciferase assays (DLA) were described previously (6). Briefly, *MYD88* isoforms (1-100 ng), NF-κB firefly luciferase reporter (100 ng) and Renilla luciferase control reporter (10 ng) were transfected into HEK293T cells. 48 h after transfection cell lysates were measured using the Dual-Luciferase Reporter Assay System by Promega according to instructions. Further details in **Supplemental Information**.

### Immunoprecipitation, SDS-PAGE and Immunoblot

For immunoprecipitation cell lysates (RIPA buffer with phosphatase and protease inhibitors) were incubated for 1.5 h with MyD88 D80F5 (CST) or anti-GFP G1544 as control antibody (Sigma), then protein G dynabeads (Thermo Fisher) were added for 1.5 h at 4°C. Beads were washed 3 times with RIPA buffer and the bound proteins were eluted with 2X LDS sample buffer (Thermo Fisher). For immunoblot cell lysates or elutions were separated on 10% or 4%–12% SDS-PAGE gels. Proteins blotted onto nitrocellulose membranes were probed with anti-HA H3663 (Sigma-Aldrich), MyD88 4D6 (epitope surrounding Leu77, Thermo Fisher), MyD88 D80F5 (epitope surrounding Cys233, CST) and MyD88 3699 (epitope surrounding Lys119, CST), HRP-conjugated secondary antibodies (1:8000) or HRP-conjugated anti-Mouse IgG (1:1000, Kappa light chain) and visualized using CCD-based ECL detection. Further details in **Supplemental Information**.

### Quantitative PCR

Upon total RNA isolation (RNeasy Mini Kit, Qiagen) and reverse transcription, qPCR reactions (20 ng cDNA, 0.3 or 1 µM primers, 1x FastStart Universal SYBR Green Master Rox, Sigma; Germany) were performed and normalized to GAPDH expression. Primer sequences and concentrations are shown in **Table S1**. Further details in **Supplemental Information**.

### Lymphoma, CLL and Ovarian Cancer Dataset Analysis

RNAseq libraries for Burkitt’s Lymphoma (BL, n=20), Follicular Lymphoma (FL, n=80), Diffuse Large B cell Lymphoma (DLBCL, n=71), FL-DLBCL (n=15), naïve B cells (n=5) and germinal center B cells (n=5) were from the European genome-phenome database archive at EBI: https://www.ebi.ac.uk/ega/home. Chronic Lymphocytic Leukemia (CLL) RNAseq data (n=289) from the ICGC-CLL Consortium (https://dcc.icgc.org/releases) (29, 30). Ovarian cancer RNAseq libraries (n=85) were from the ICGC/OV-AU project (Australian Ovarian Cancer Study, https://dcc.icgc.org/projects/OV-AU) (31, 32). For RNAseq data analysis, isoform 2 abundance was calculated as 1-Σ_isoforms_, because it has no unique splice site, intron retention (isoform 8) was calculated as relative to the flanking exons’ expression and the relative usage of exon 4 acceptor splice site. For the rest of isoforms, the number of unique splice junctions divided by number of reads at the respective splice site is shown. The unique splice junctions considered for analysis are for isoform 1: exon 3 + 20nt ➔ exon 4, isoform 3: exon 1 ➔ exon 3, isoform 4: exon 2 ➔ exon 4, isoform 5: exon 1 ➔ exon 4, isoform 6 and 7: exon 3 -20nt ➔ exon 4. More details are given in **Supplemental Information**.

### Statistic Analysis

Experimental data was analyzed using Excel 2010 (Microsoft) and/or GraphPad Prism 6, 7 or 8 or in *R*, flow cytometry data with FlowJo 10. Normal distribution in each group was always tested using the Shapiro-Wilk test first for the subsequent choice of a parametric (ANOVA, Student’s t-test) or non-parametric (e.g. Friedman, Mann-Whitney U, Kruskal Wallis or Wilcoxon) test. p-values (α=0.05) corrected for multiple testing were then calculated in Prism. Values <0.05 were generally considered as statistically significant and denoted by * or # throughout. Comparisons were made to unstimulated control, unless indicated otherwise, denoted by brackets.

## Results

### *MYD88* Displays Comprehensive Splicing Leading to Functionally Disparate Isoforms

Given the importance that the MyD88s splice variant has been ascribed in murine myeloid cells (17, 23), we sought to conduct a systematic characterization of all known human *MYD88* splice variants. Until recently, five *MYD88* mRNA transcripts with differential splicing have been reported (**Table 1** and **Figure 1A**), giving rise to five protein isoforms with different domain structure (**Figure 1B**). Compared to the canonical isoform 2, isoform 1 features an additional 8 amino acids in frame between exon 3 and 4, i.e. in the TIR domain, due to the use of an alternative splice site (dark grey box and/or dashed lines in **Figures 1B** and **S1B**). Isoform 3 lacks the ID (exon 2) but includes both DD and TIR domain and corresponds to the aforementioned MyD88s variant. Isoform 4 and 5 both lack the TIR domain entirely, due to frame-shifts resulting from the skipping of exon 3 (**Figure S1A**). In terms of canonical MyD88 domains, isoform 4 thus is limited to a DD-ID protein followed by 36 C-terminal amino acids that bear no apparent similarity to any known proteins (**Figure S1A**). In isoform 5, exon 2 is additionally skipped, thus resulting in a DD-only variant. In order to investigate functional differences, these isoforms were cloned into StrepHA-tagged expression constructs and their expression verified in transfected HEK293T cells. Evidently, all constructs could be detected as proteins of 40, 37, 35, 27 and 23 kDa (**Figure 1C** and **Table 1**), albeit with different expressions levels. The shortest isoform, termed isoform 5, was barely detectable, indicating it may be less stable. Next, we assessed the ability of all isoforms to drive NF-κB activation using dual luciferase assays upon transfection of equal amounts of expression plasmids in HEK293T cells. Whilst this assay cannot report on the ability to transduce incoming TLR signals, it is well established to assess MyD88 downstream signaling potential (2, 6, 7, 33–35). Here, isoform 1 was the most active isoform, followed by isoform 2, the canonical MyD88 splice variant (**Figure 1D**). Isoform 4 was also able to induce NF-κB activity, at slightly lower levels. Isoform 3 and 5 were not able to induce NF-κB activity, consistent with a lack of ID, which is required to assemble into a Myddosome and recruit IRAK4 (4, 34). Since HEK293T cells endogenously express MyD88 isoform 2 at high levels (*cf.* **Figure 1C**), we also conducted the experiment in the MyD88-deficient HEK293T-derivative cell line, I3A (33). An almost identical picture emerged, where the canonical isoform 2 induced the highest NF-κB activity (**Figure 1E**). Since both murine and human MyD88s (isoform 3) were described as dominant-negative regulators of canonical MyD88 due to lack of the ID (34, 36), we also tested whether isoforms 3 and 5 could block TLR signaling, e.g. *via* TLR5, in the HEK293T system, but this was not the case (**Figures S1C, D**). Collectively, non-canonical MyD88 isoforms with an intact DD and ID (isoforms 1 and 4) are capable of transmitting downstream NF-κB activity and their expression may thus support the function of the canonical MyD88 (isoform 2), whereas isoforms 3 and 5 are inactive.

### Primary B Cells Express Multiple *MYD88* Splice Isoforms

All analyses on *MYD88* splicing have so far focused on (mostly transformed) myeloid and epithelial cells but as aforementioned MyD88 also plays an oncogenic role in B cells *via* NF-κB signaling (11). To assess the expression levels of these isoforms in primary B cells and be able to identify them by molecular weight *via* SDS-PAGE, we also generated expression constructs without a tag as a ‘molecular ladder’. Whole cell lysates from HEK293T transfected with these untagged isoforms 1 to 5 and from unmodified and MyD88 knockout reporter THP-1 cells (see Methods) were then compared alongside lysates of primary B cells from two different healthy donors. We could detect the expression of four different MyD88 isoforms, identifying isoforms 2, 3 and (probably) 4 as matching the molecular weight of the untagged expression constructs and strongly reduced or absent in the edited THP-1 cells (**Figure 1F**). In long exposures a band migrating at the height of isoform 5 was also visible in 1 donor but not THP-1 cells. Collectively, the canonical isoform 2 shows the highest protein expression levels in primary B cells and isoform 5 the lowest (**Figure 1F**).

### Transformed B Cells Also Express Multiple *MYD88* Splice Isoforms

**Figure 2:**
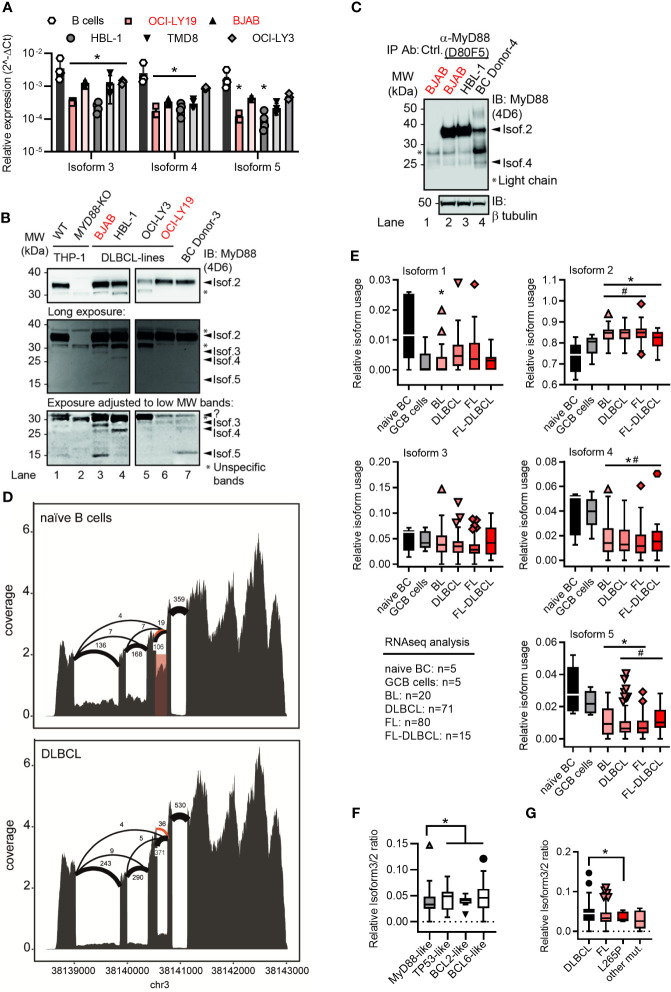
Lymphoma cell lines and primary tumor samples show a preference for the canonical *MYD88* isoform. **(A)** RT-qPCR analysis of isoforms 3-5 in primary B cells or lymphoma cell lines (n=3-4; red GCB, black ABC). **(B)** Immunoblot from THP-1 myeloid cells, B lymphoma cell lines and primary B cells (n=3). **(C)** Immunoblot from pulldown of MyD88 isoforms using the antibody D80F5 against the TIR domain (exon 4). **(D)** Sashimi plots with mean read numbers supporting the splice junctions from naïve B cells (n=5) and DLBCL samples (n=83). The red shaded box shows intron retention and orange arcs represent an alternative donor splice site from isoforms 6 and 7. **(E)** RNAseq analysis of relative isoform usage from untransformed B cells or lymphoma samples (n=as indicated). Isoform 2 expressed as 1-(sum of all others). Other isoforms used: number of unique splice junctions divided by number of reads at the respective splice site (see Methods). **(F, G)** Ratios of isoform 3 (*MYD88s*) to isoform 2 in different DLBCL sub-clusters **(F)** and in dependence of *MYD88* mutations **(G)**. **(F)** MyD88-like n=24, BCL2-like n=9, BCL6-like n=16 and TP53-like n=19. **(G)** MyD88 L265P mutated samples (n=5) and other mutants (n=6) compared to respective MyD88 wildtype lymphomas. **(A, E–G)** represent combined data (mean+SD, or Tukey box and whiskers) from ‘n’ biological replicates (each dot represents one replicate). In B one representative of ‘n’ technical replicates is shown. * or # = p<0.05 according to two-way ANOVA **(A)**, Mann-Whitney (**E**, comparison to naïve B cells (*) or to GCB cells (#)), or Wilcoxon **(F, G)**.

As expression patterns between primary and transformed cells may differ, we next characterized the expression of the five isoforms in several ABC and GCB DLBCL cell lines using isoform-specific primers to distinguish isoforms 1/2 from other isoforms (**Figures S2A, B**, Methods and **Table S1**). This confirmed the expression of isoforms 3, 4 and 5 at mRNA level in these cell lines (**Figure 2A**). Using lysates of these ABC and GCB cell lines and an antibody directed against the DD, multiple MyD88-specific bands were also detectable (**Figure 2B**). Taking into account the predicted molecular weights of the alternative isoforms (*cf.* **Table 1** and **Figure 1F**) and their corresponding mRNA levels in BJAB cells *vs* primary B cells (*cf.* **Figure 2A**), certain labeled bands in **Figure 2B** are likely to correspond to isoform 3, 4 and 5. This same pattern of bands was observed using a combination of 2 additional anti-MyD88 antibodies (**Figure S2C**). To enrich the alternative isoforms from whole cell lysates, we pulled down MyD88 using an antibody, which is directed against the TIR domain (exon 4) and thus should detect isoforms 1, 2 and 3. Subsequent immunoblot of the elution showed bands corresponding to isoform 2 and surprisingly isoform 4, possible due to DD-mediated heterodimer formation (6) with isoform 2 (**Figure 2C**). Any detected alternative isoforms were less prominent than isoform 2 (**Figure 2B, C**) in the DLBCL lysates. This suggests that B cells express multiple MyD88 splice isoforms both on mRNA and protein level but isoform 2 is also dominant in transformed B cells.

### Primary B Cell Malignancies Show a Preference for Isoform 2

As these transformed cell lines may not reflect primary tumors, we next characterized the RNA expression of the five isoforms in primary B cell lymphoma samples and untransformed naïve B cells. Sashimi plots of RNAseq data from a total of 186 different lymphoma cases (Burkitt lymphoma, DLBCL, follicular lymphoma, follicular lymphoma-DLBCL), untransformed germinal center B cells (GCB, n=5) and naïve peripheral blood B cells (n=5, acquired by the German ICGC MMMLSeq consortium, see Methods) showed expression of all five isoforms at mRNA level (**Figures 2D** and **S2D**). Consistent with earlier mRNA and protein analysis, the canonical isoform 2 was significantly more abundant in transformed *vs* untransformed B cells, whereas other isoforms were either comparable between these groups (isoform 3) or significantly lower (isoform 1, isoform 4 and isoform 5) (**Figure 2E**). Thus, transformed primary B cell tumor samples also showed a preference for the canonical isoform 2 – but not isoform 3 (MyD88s) or other non-canonical isoforms. This was surprising as an ‘NF-κB signature’ has been attributed to these types of entities (14–16) and in myeloid cells NF-κB signaling was proposed to induce MyD88s (isoform 3) as aforementioned. Collectively, this suggests that, contrary to expectations, lymphoma samples show a higher ratio of canonical MyD88 (isoform 2) to MyD88s (isoform 3) than naive B cells. The analysis of sub-clusters (dependent on driver mutations) of DLBCL samples suggested that those driven by direct activators of NF-κB signaling (e.g. an *‘*MyD88-like’ sub-cluster, see Methods) had a lower ratio of alternative splicing *vs* canonical, and specifically isoform 3, than those driven by indirect NF-κB activation (e.g. BCL2-, BCL6- and TP53-like DLBCL, see **Figures 2F** and **S2E**). In line with this, samples with NF-κB-promoting *MYD88* gain-of-function mutations, such as L265P, had a lower isoform 3 *vs* isoform 2 ratio, i.e. expressed significantly more isoform 2 *vs* isoform 3 transcripts (**Figure 2G**). At least on mRNA level, primary B cell tumors thus did not show evidence for an isoform 3-mediated negative feedback look despite an ‘NF-κB signature’ described for these entities.

### TLR Stimulation Induces Isoform 3 Only Transiently in Stimulated B Cells

**Figure 3:**
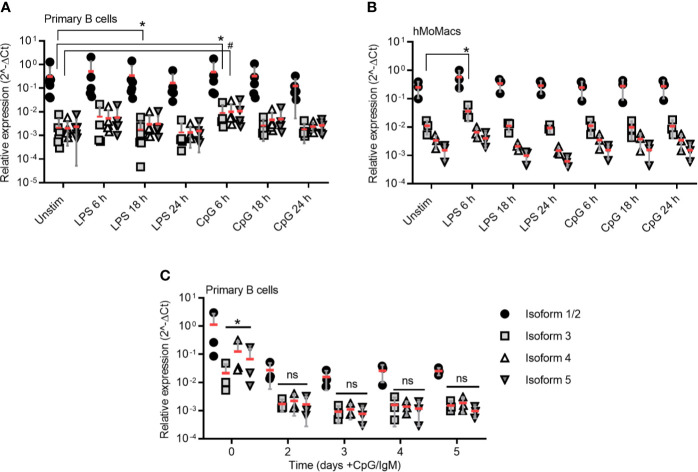
TLR stimulation induces isoform 3 only transiently in primary B cells. **(A–B)** RT-qPCR analysis of isoforms 1/2 to 5 in primary B cells (**A**, n=5-7) and hMoMacs (**B**, n=3) stimulated with LPS or CpG as indicated (n=3). **(C)** RT-qPCR analysis of isoforms 1/2 to 5 in primary B cells stimulated with CpG and IgM to induce proliferation (n=3). A-C represent combined data (mean+SD from ‘n’ biological replicates (each dot represents one replicate). ns, non-significant; * or # = p<0.05 according to Kruskal-Wallis test **(A)**, ordinary one-way ANOVA **(B)** or two-way ANOVA **(C)**.

Based on what has been published regarding the induction of MyD88s *via* NF-κB signaling in myeloid cells (17, 36), we next tested whether defined NF-κB activating stimuli, e.g. LPS for TLR4 and CpG for TLR9, would lead to an upregulation of isoform 3 in freshly purified (**Figure S3A**) primary B cells. Indeed, TLR9 stimulation enhanced mRNA levels of isoform 3 and 4 at 6 h (mean fold change = approx. two-fold), but at later time points it decreased again to unstimulated levels. TLR4 stimulation induced a marginal but significant reduction of isoform 3 at 18 h (**Figure 3A**). Overall, TLR stimulation changed the relative ratios of *MYD88* splice isoforms very little and the variability between donors is high. As control, we isolated, differentiated and stimulated hMoMacs from the same donors and observed an increase upon 6 h TLR4 stimulation, in line with earlier studies (**Figure 3B**), although it has to be borne in mind, that these earlier studies mainly tested in murine macrophages or human epithelial cells (17, 26, 36). Conversely, when B cells were stimulated until proliferation with TLR9 CpG + IgM, surprisingly, *MYD88* transcription was reduced altogether and did not lead to higher relative induction of the MyD88s (isoform 3, **Figure 3C**), despite the fact that TLR stimulation was effective at driving cellular proliferation as assessed by CFSE proliferation assays (**Figure S3B**). Therefore, we conclude that proliferating B cells, like lymphoma samples, show and maintain a preference for canonical MyD88 signaling. Furthermore, in B cells sustained NF-κB signaling does not induce or coincide with a shift towards inhibitory isoforms as reported for myeloid cells regarding MyD88s (isoform 3). Rather, the canonical, signaling-competent isoform 2 dominates

### Novel MyD88 Isoforms With TIR Truncation in B Cells Are Supportive of NF-κB Signaling

**Figure 4:**
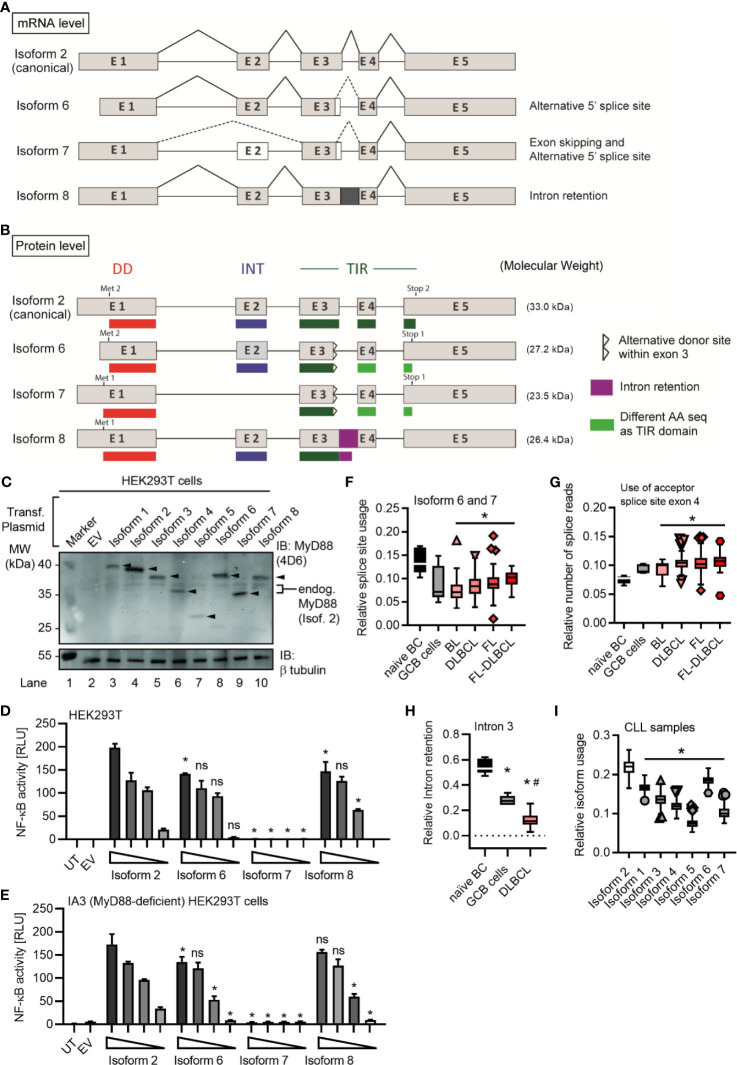
*MYD88* can give rise to three additional MyD88 isoforms. **(A, B)** Schematic representation of novel *MYD88* isoforms on mRNA and protein level according to references in **Table 1** and hypothetical sequence for isoform 8. **(C–E)** HEK293T cells were transfected with plasmids for different *MYD88* isoforms and lysates analyzed for expression or pathway activation by immunoblot (**C**, n=2) or NF-κB dual luciferase assay (**D, E** n=3), respectively. **(E)** as in D but using MyD88-deficient I3A cells (n=3). **(F–H)** RNAseq analysis from untransformed B cells or lymphoma samples (n=as indicated in **Figure 2E**). Intron retention presented as relative number of splice reads using the acceptor splice site of exon 4 **(G)** or coverage of intron 3 compared to mean of flanking exons 3 and 4 **(H)**. **(I)** RNAseq analysis from CLL samples (n=289). In **C–E** one representative of ‘n’ technical replicates is shown, for **D, E**, as mean + SD from three repeats. **F–I** represent combined data (Tukey box and whiskers) from ‘n’ biological replicates (each dot represents one replicate). ns, non-significant; * or # = p<0.05 according to two-way ANOVA comparing to isoform 2 **(D, E)** or Wilcoxon Mann-Whitney **(F-I)** in comparison to naïve B cells (*, **F–H**) and to GCB cells (#, **F–H**) or isoform 2 **(I)**.

In the process of RNAseq analysis we noticed additional alternative splicing events, namely either usage of another donor splice site within the exon 3 (leading to isoforms 6 and 7) or the retention of the exon 3-4 intron (here termed isoform 8), see **Figures 2D**, **4A, B**, **Figures S4A, B** and **Table 1**. The novel splice site within exon 3 (20 nt upstream of a canonical donor) showed a Human Splicing Finder (HSF) score of 81. Typically, a score above 65 is considered a strong splice site (37), indicating these additional splicing events are highly plausible. This alternative donor site leads to a premature STOP codon and thus results in additional isoforms with a truncated TIR domain (**Figures 4A, B** and **Figures S4A, B**), which have not been reported so far. When expression constructs corresponding to isoforms 6-8 were transfected into HEK293T cells, proteins of the expected size (29 kDa for isoform 6, 24 kDa isoform 7 and 26 kDa for isoform 8; plus 6 kDa from the StrepHA-tag) were detectable (**Figure 4C** and **Table 1**). The isoform 8 construct was generated from an hypothetical sequence, which was confirmed by sequencing BJAB amplification product upon PCR using specific primers (**Table S1** and **Figure S4B**). To gain an insight into their ability to signal to NF-κB, we performed NF-κB dual luciferase assays in normal HEK293T and I3A cells as before. Evidently, isoforms 6 and 8 were able to induce downstream NF-κB activation in HEK293T cells, whereas isoform 7 did not (**Figures 4D, E**).

Isoform 6-8 transcripts were also detectable in the lymphoma samples (**Figures 4F–H**) and, as with the other non-canonical isoforms, they were significantly less abundant in lymphoma cells *vs* naive B cells. In the 289 RNA-seq samples of the ICGC Chronic Lymphocytic Leukemia (CLL) dataset, 7 isoforms could be readily detected and quantified, with the canonical isoform showing the highest relative abundance, followed by isoform 6, while isoform 5 showed the lowest abundance (**Figure 4I**). Furthermore, there were noticeable reads mapping to the exon 3-4 intron (**Figure S4C**) confirming isoform 8 in CLL. Additionally, we could also detect isoform 8 in primary B cells (**Figure S4D** and hMoMacs (**Figure S4E**) by RT-qPCR. Interestingly, TLR4 stimulation in hMoMacs significantly enhanced the mRNA levels of isoform 8, another signaling competent form (cf. **Figures 4D,E**). All eight *MYD88* splice isoforms were also detectable in non-immune cells, as verified in a publicly available RNAseq dataset (31) for ovarian cancer (**Figure S5**). On the whole, there are 3 additional splice isoforms of MyD88 with truncated TIR domains out of which two, unexpectedly, can support signaling upon overexpression, similar to the canonical MyD88 isoform. This extended analysis highlights an even higher diversity of splice variants emanating from the *MYD88* oncogene than previously thought. Furthermore, splicing in B cell lymphomas appears to strongly favor the canonical *MYD88* isoform without diverting splicing events to alternative or signaling-incompetent splice isoforms. Importantly, we find no evidence for a significant induction of MyD88s (isoform 3) as a restrictor of TLR pathway activity.

## Discussion

Alternative splicing has emerged as a frequent phenomenon employed for fine-tuning or regulating signaling pathways and plays a pivotal role in the adaptive immune system (38, 39). However, decisive regulators of innate immune pathways have also been subject to alternative splicing: Since its discovery in 2002, the induction of MyD88s *via* NF-κB signaling loop has been viewed as a classical example of an inflammation-restricting negative feedback loop in innate immunity (17, 27). Hence, all the numerous subsequent studies on MyD88 splicing have exclusively focused on this isoform (23, 24, 40–42) and have been largely limited to myeloid cells, primarily in the murine system.

We here provide a comprehensive characterization of all currently reported human *MYD88* splice isoforms. This includes the novel isoforms 6-8, which are the only variants to contain partial TIR domains. During the course of this analysis, isoforms 6 and 7 were added to Genebank but had not been confirmed or studied in detail. Isoform 8 is a novel and surprisingly frequent splicing event not reported before and found abundantly in naïve B cells. Our analysis suggests that, with the exception of isoforms 3 (MyD88s), 5 and 7, isoforms (4, 6 and 8) may induce downstream NF-κB activity in overexpression assays. Whether they can nucleate or engage in the Myddosome in response to TLR signaling in the absence of a complete TIR domain remains to be studied. Potentially, isoforms 4, 6 and 8 may also be signaling incompetent. Thus, all *MYD88* splice isoforms, except isoforms 1 and 2, may lead to dysfunctional MyD88 proteins. This would make our observations made on transcript levels even more striking as then none of the alternative splicing events would be able to counteract constitutive NF-κB signaling *via* isoform 2. Consequently, the oncogenic influence of isoform 2 is likely to be even more dominant.

**Figure 5:**
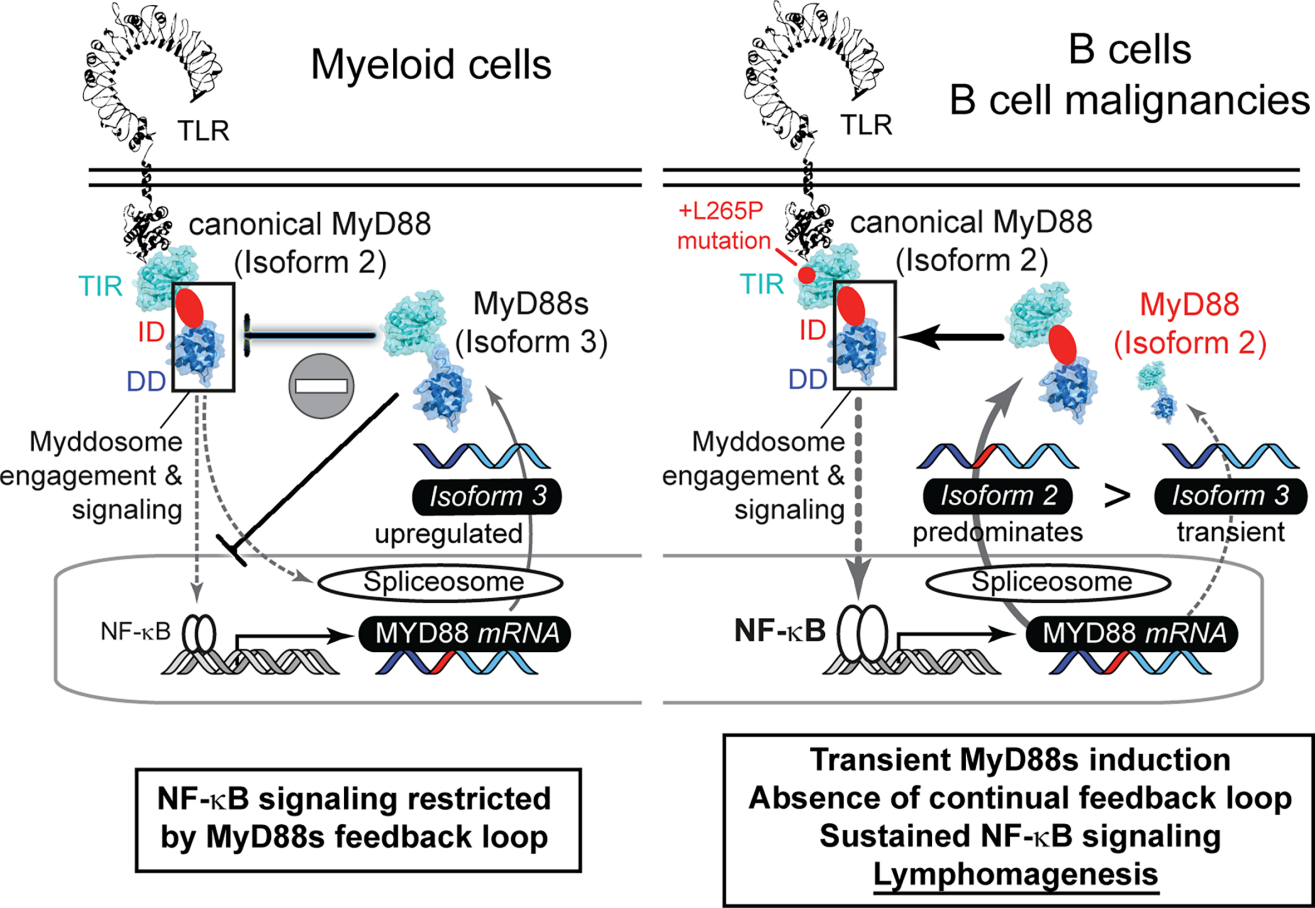
The failure of proliferative B cells to induce non-canonical *MYD88* splice variants correlates with lymphomagenesis *via* sustained NF-κB signaling. Graphical abstract summarizing splicing patterns in myeloid cells (left, based on literature) and B cells (right, focus in this study).

Furthermore, we show that *MYD88* splicing is much more multi-faceted than previously reported: Our data indicate that whereas normal B cells use a richer repertoire of splice isoforms, the transformed status rather displays a reduced diversity and appears to lack alternative splice events. The reason for this is unknown but our data warrant a further investigation in additional cohorts and entities, e.g. Waldemström’s macroglobulinemia, in future. Based on our data it appears that the preference for canonical isoform 2 and thus unrestricted NF-κB signaling may be favored in the oncogenic process. BCL2, BCL6 or TP53-driven lymphomas, which have an indirect effect on the NF-κB signature, showed lower levels of canonical *MYD88* and higher levels of isoform 1 and isoform 4, compared to MyD88-like lymphomas (**Figures 2F** and **S2E**). This fits well with the observation that the gain-of-function mutation, L265P, leads to extended NF-κB hyperactivation and is a hallmark of oncogenic B cells (7, 8). Of note, our data indicate that B cells lack a sustained negative feedback mechanism of MyD88s induction to rescue mutated cells from MyD88-driven oncogenesis: For example, TLR stimulation induced MyD88s in TLR-stimulated hMoMacs and B cells at short time points, but MyD88s was not prominently expressed or regulated under the extended presence of NF-κB stimuli in B cells and lymphoma cell lines. Thus, B cells with increased NF-κB activity, due to L265P mutation or other mechanisms, cannot get “reigned in” (controlled) *via* MyD88s expression, unlike some myeloid cells, then continued NF-κB pro-survival activity may result (**Figure 5**). Our data thus provide an explanation why oncogenic mutations have only been reported in B cell lymphoma, rather than tumors arising from myeloid cells, whose MyD88s induction loop possibly renders them more resistant to MyD88 pathway induced NF-κB activity.

Our observations that alternative splicing of genes in the MyD88 dependent pathway are important candidates in oncogenesis agree with the recent description of oncogenic *IRAK4* isoforms, albeit in myeloid malignancies (43). It is intriguing to speculate whether the aforementioned negative feedback loop, that is absent in proliferative B cells, prevents *MYD88* mutations from manifesting themselves, but does not prevent oncogenic signaling arising from the next downstream pathway member, IRAK4. Undoubtedly, with the availability of powerful sequencing techniques the analysis of alternative splice isoforms of MyD88 pathway members for discovering novel non-mutational cancer drivers is both possible and warranted. In the substantial percentage of cases without druggable driver mutations this may offer opportunities for targeting e.g., *via* antisense oligonucleotide-mediated exon skipping (44, 45). In this therapeutic sense, MyD88s or the other signaling incompetent isoforms described here may provide a blueprint for such an approach in B cell lymphomas.

## Data Availability Statement

Publicly available datasets were analyzed in this study. B cell lymphoma, naive B cells and Germinal center B cells RNAseq libraries are from European genome-phenom archive at EBI: https://www.ebi.ac.uk/ega/home. Chronic Lymphocytic Leukemia (CLL) RNAseq data from the ICGC-CLL Consortium (https://dcc.icgc.org/releases). Ovarian cancer RNAseq libraries from the ICGC/OVAU project (Australian Ovarian Cancer Study, https://dcc.icgc.org/projects/OV-AU).

## Ethics Statement

The studies involving human participants were reviewed and approved by Ethics committee of the Medical Faculty, University of Tübingen. The patients/participants provided their written informed consent to participate in this study.

## Author Contributions

YC, O-OW and SD performed experiments. YC, SB, SF, SN, SD, JA, and SO analyzed data. RS and SO were involved in sample collection. YC and AW conceived and AW supervised the entire study. YC and AW wrote the manuscript and all authors provided additions and comments to the manuscript. All authors contributed to the article and approved the submitted version.

## Conflict of Interest

The authors declare that the research was conducted in the absence of any commercial or financial relationships that could be construed as a potential conflict of interest.

## Abbreviations

BL, Burkitt Lymphoma; CLL, Chronic Lymphocytic Leukemia; DD, Death Domain; DLA, Dual Luciferase Assay; DLBCL, Diffuse Large B Cell Lymphoma; FL, Follicular Lymphoma; GCB, Germinal Center B Cells; HEK, Human Embryonic Kidney; ID, Intermediate Domain; IL-1R, Interleukin-1 Receptor; IRAK, IL-1R-Associated Kinase; LPS, Lipopolysaccharide; Myd88, Myeloid Differentiation 88; NF-κb, Nuclear Factor κb; TIR, Toll/Interleukin-1 Receptor; TLR, Toll-Like Receptor; TNF, Tumor Necrosis Factor; WT, Wild-Type.
