# Supplementary material for: Absence of Non-Canonical, Inhibitory MYD88 Splice Variants in B Cell Lymphomas Correlates With Sustained NF-κB Signaling
Source: Front Immunol. 2021 Jun 7;12:616451. doi: 10.3389/fimmu.2021.616451 (PMC8215704; doi:10.3389/fimmu.2021.616451)
Supplement: Supplementary file 1 [file DataSheet_1.docx]

**Supplementary Material**

## Supplemental Methods

#### Isolation and stimulation of primary human immune cells

Peripheral blood mononuclear cells (PBMCs) from healthy donors were isolated from whole blood or buffy coats (University Hospital Tübingen Transfusion Medicine) using Ficoll density gradient purification. Primary B cells were isolated from PBMCs by negative selection using B Cell Isolation Kit II (Miltenyi Biotec; Germany) according to instructions. B cell isolation always reached >90% purity and cells were seeded in supplemented RPMI (Life Technologies; CA, USA) with 10% human serum and rested for at least 4 h before the experiment. Primary monocytic derived macrophages (hMoMacs) were isolated from PBMCs using Monocyte attachment Medium (PromoCell) according to instructions and monocytes were differentiated for 6 days in supplemented RMPI with 10% FCS + 50 ng/ml hGM-CSF (Sanofi) to macrophages. 2 x10^6 B cells or 1x10^6 hMoMacs were stimulated for 0, 6, 18 and 24 h with 2.5 µg/ml CpG 2006 (TIB MOLBIOL; Germany) or 200 ng/ml LPS (from *E. coli* K12, Invivogen; CA, USA). For RT-qPCR measurements cells were lysed in RLT buffer + β-mercaptoethanol (Qiagen; Germany). To track cell proliferation, 5 x 10^6 B cells were stimulated for 0, 2, 3, 4, 5 days with 2.5 µg/ml CpG 2006 and 5 μg/mL anti-human IgM (Fc5µ, Jackson Immuno Research; PA, USA) and processed for RT-qPCR, or cells were additionally stained on day 0 with carboxyfluorescein-succinimidyl ester (CFSE, Life Technologies; CA, USA). On day 5, proliferative cells were washed and stained with anti-CD19 Pacific Blue (Biolegend; CA, USA), then cells were analyzed on a BD FACSCanto™ II system with 488 nm excitation for CFSE and 405 nm for Pacific Blue. Graphs were generated using software FlowJo PC version 10.

#### Plasmid constructs

*MYD88* isoform expression constructs were generated in pTO-N-SH vector using the Gateway cloning system (Thermo Fisher; MA, USA) adding a fused StrepIII-Hemagglutinin tag at the N-terminus of the gene of interest. Additionally, Isoforms 1 to 5 were generated in pTO-C-SH vector, in which the coding sequences (CDS) include a stop codon to not translate the tag. The CDS of MyD88 isoform 1 to 7 were taken from reference sequences listed in Table 1. For *MYD88* isoform 8, canonical isoform 2 sequence was taken as template and the corresponding intron sequence between exon 3 and 4 was added. *MYD88* isoform 1 was purchased from Harvard Plasmids (HsCD00296025) and isoform 2 was described earlier (Avbelj et al., 2014)[. Other CDS were synthesized by the company Genewiz and verified by DNA sequencing.](#_ENREF_7) Illustrations were generated using Geneious 5.5.9 and Adobe Illustrator.

#### Cell cultures

All DLBCL cell lines were described previously(Avbelj et al., 2014)[. DLBCL cell lines were cultured in RPMI supplemented with 20% heat-inactivated fetal bovine serum (FBS, Gibco), except OCI-LY19 (Minimum Essential Medium Alpha, 10% FBS, life technologies). HEK293T cells were cultured in Dulbecco’s modified Eagle medium DMEM (Invitrogen; France) supplemented with 10% FBS. THP-1 WT and MyD88 deficient cells (a gift from R. Amann, University of Tübingen, Germany) were cultured in RPMI with 10% FCS.](#_ENREF_7)

#### Dual Luciferase Assay

For dual luciferase assays (DLA) 75,000 HEK293T WT or MyD88-deficient I3A cells were plated on a 24-well format and transiently transfected with plasmids expressing *MYD88* isoforms (1-100 ng), firefly luciferase under the NF-κB promoter (100 ng) and Renilla luciferase under SV40 promoter (10 ng). The total amount of plasmid was adjusted with the empty vector. 48 hours after transfection cells were lysed in passive lysis buffer (Promega; WI, USA) and lysates were measured for luciferase activity on a FluoStar luminescence plate-reader (BMG Labtech). Analysis settings were chosen as recommended in the Dual-Luciferase Reporter Assay System by Promega using MARS data analysis software version 1.20. Graphs and statistics were done in GraphPad Prism version 8.

#### SDS-PAGE and immunoblot

#### To check expression of MyD88 endogenous proteins and proteins derived from plasmids immunobloting was performed. Cells were lysed in RIPA buffer (20 mM Tris-HCl pH 7.4, 150 mM NaCl, 1 mM EDTA, 10% glycerol, 0.1% SDS, 1% Triton X-100 and 0.5% deoxycholate) supplemented with PhosSTOP, EDTA-free protease inhibitor cocktail (both from Roche) and 0.1 μM PMSF. Reduced and denatured whole cell lysates (WCL) from transient transfected HEK293T cells were separated on 10% Tris-glycine gels using SDS running buffer (25 mM Tris-base, 250 mM glycine and 0.1% SDS) and WCLs used to test endogenous protein were run on 4%–12% gradient gels using MOPS running buffer (Invitrogen). Separated proteins were transferred onto nitrocelullose membranes (GE Healthcare, 0.45 µm) for 35 min at 25 V. Then, membranes were blocked 1h at room temperature in 5% milk in Tris-buffered saline solution with 0.1% (vol/vol) Tween-20 (TBS-T) and were probed overnight with primary antibodies (all diluted 1:1000): anti-HA H3663 (Sigma-Aldrich; Germany), anti-beta-Tubulin 2A1A9 (abcam; UK), MyD88 4D6 (Thermo Fisher), MyD88 D80F5 and 3699 (CST; MA, USA). Next day HRP-conjugated secondary antibodies, anti-rabbit (Biozol; Germany) and anti-mouse (Promega), were applied at 1:8000 dilution for 2 h. Optimal conditions to visualize the mayority of the alternative isoforms were used for Fig. 1D and 2C, consisting of 10% Bis-Tris gels, MOPS running buffer, 30 min 25V for the transfer and membranes were blocked with Intercept Blocking buffer (Lycor). Additionally, the amplifier HRP-conjugated anti-Mouse IgG (1:1000, Kappa light chain) was used. All membranes were washed three times for 5 min with TBS-T after each antibody incubation. Detection was done by chemiluminescence (Peqlab) and development using a charge-coupled device camera or Odyssey FC Imager to capture the luminescent signals. Pictures were analyzed and edited in Phusion (Peqlab), Image Studio ver 5.2 (LyCor) and Adobe Illustrator programs.

#### Quantitative PCR

#### Total RNA isolation was performed by a Qiacube robot using reagents from the RNeasy Mini Kit from Qiagen including DNA digestion (RNase-Free DNase Set, Qiagen). mRNA transcription to cDNA was done manually using High Capacity RNA-to-cDNA (Thermo Fisher). Quantitative PCR was performed in reactions containing 20 ng cDNA, 0.3 or 1 µM of primers, 1x SYBR Green (FastStart Universal SYBR Green Master Rox, Sigma) and RNA-free water. Primers (Table S1) were designed to discriminate all the tested MYD88 isoforms and map to the exon junctions fulfilling compatibility requirements with SYBR Green mix, which were evaluated in the publicly available software Primer3 (http://bioinfo.ut.ee/primer3-0.4.0/). Of note primers detecting isoform 2 simultaneously amplify isoform 1 (see Fig. S2C); nevertheless isoform 1 suggested to have very low abundance (data not shown) and melting curves show mainly one peak corresponding to isoform 2. Each sample was analyzed in triplicates in a real-time cycler (Thermo QuantStudio 7 Flex, Thermo Fisher). The cycling profile applied was: 10 min/95 °C; 40 cycles of 95 °C/15 s and 60 °C/1 min, followed by a continuous melt curve stage from 50°C to 95°C. Data was analyzed with the QuantStudio 6 and 7 Flex software and normalized to GAPDH expression.

#### Lymphoma dataset analysis

#### B cell lymphoma RNAseq libraries from 190 samples, including Burkitt’s Lymphoma (BL, n=21), Follicular Lymphoma (FL, n=83), Diffuse Large B cell Lymphoma (DLBCL, n=72), and FL-DLBCL (n=14) were acquired by the German ICGC MMMLSeq consortium and were uploaded as part of several publications to the European genome-phenom archive at EBI: https://www.ebi.ac.uk/ega/home. Naïve B cells (B cells, n=5) and germinal center B cells (GC B cells, n=5) libraries were used as control data and were made public in the same way. Details for library preparation can be found under related papers (Richter et al., 2012;Kretzmer et al., 2015;Hezaveh et al., 2016;Lopez et al., 2019). To visualize alternative splicing events, RNA sequencing data was mapped onto the human reference genome hg38 (UCSC genome) using Segemehl version 2.0 alpha (Hoffmann et al., 2014). Splice reads overlapping with the human MYD88 gene were counted and visualized in Sashimi Plots (Katz et al., 2015) using R's ggplot2. Also a compositional data approach used in the DIEGO software (Doose et al., 2018) was applied to analyze differential splicing patterns of the MYD88 gene. The support number of every splice junction is considered relative to all splice junctions of the MYD88 gene, and possible variations are analyzed using Wilcoxon’s rank sum test as implemented in R. Isoform 2 abundance was calculated as 1- sum of all other splice sites abundances, because it has no unique splice site. Intron retention was calculated as mean intron coverage relative to the mean coverage of the two flanking exons (Broseus and Ritchie, 2020) and the relative usage of exon 4 acceptor splice site. Furthermore, the unique splice junctions considered for analysis are for Isoform 1: exon 3+20nt 🡪 exon 4, Isoform 3: exon 1 🡪 exon 3, Isoform 4: exon 2 🡪 exon 4, Isoform 5: exon 1 🡪 exon 4, Isoform 6 and 7: exon 3 -20nt 🡪 exon 4. DLBCL sub-cluster classification was performed by the ICGC MMML-Seq (https://icgc.org/node/53049; Hübschmann et al, personal communication (MyD88-like n=24 , BCL2-like n=9, BCL6-like n=16 and TP53-like n=19). MyD88 mutations (n=6) M232T, V217F, S219C, I220T, S222R, S243N and T249P were considered as gain-of-function according to Refs. (Ngo et al., 2011;Avbelj et al., 2014) apart from L265P (n=5). Generally, tumor cell purity was not adjusted,but there was no significant correlation between tumor cell content and isoform usage. Boxplot graphics were generated using GraphPad Prism version 8.

#### CLL dataset analysis

Chronic Lymphocytic Leukemia (CLL) RNAseq data from 289 patients was acquired from the ICGC-CLL Consortium (https://dcc.icgc.org/releases) and the acquisition and preparation of these libraries has been previously described (Puente et al., 2011;Ferreira et al., 2014)[. Quality of raw CLL RNA-seq data in FASTQ files was assessed using ngs-bits:ReadQC (ngs-bits version 0.1 at github.com/imgag/ngs-bits) to identify sequencing cycles with low average quality and base distribution bias. Reads were preprocessed with ngs-bits:SeqPurge](#_ENREF_47) (Sturm et al., 2016)[, mapped using STAR](#_ENREF_48) (Dobin et al., 2013) [(version 2.5.3a) to the human reference genome GRCh37 (Ensembl) and alignment quality was assessed using ngs-bits:MappingQC. Sashimi plots from CLL data were generated using the Broad Integrative Genome Viewer (IGV, version 2.3.1) to visualize splicing. To reduce false positive hits, only junctions which are covered by at least 5 reads in at least 5 of 289 analyzed samples have been retained. Splice junctions were quantified by normalizing splice junction reads with the total number of spliced reads in the](#_ENREF_49) *MYD88* gene. Normalized junction counts were then attributed to one or more matching isoforms.

*Ovarian cancer*

Ovarian cancer RNAseq libraries from 85 patient samples were acquired from the ICGC/OV-AU project (Australian Ovarian Cancer Study, https://dcc.icgc.org/projects/OV-AU). Patient cohort description and libraries preparation can be consulted in previous publications (Patch et al., 2015;O'Donnell et al., 2018). Ovarian cancer RNA sequencing data was mapped onto the human reference genome hg38 (UCSC genome) and sashimi plots were created using MISO framework version 0.5.3 (<https://miso.readthedocs.io>) (Katz et al., 2010).

#### Statistical analysis

#### Experimental data was analyzed using Excel 2010 (Microsoft) and/or GraphPad Prism 6, 7 or 8 or in R, flow cytometry data with FlowJo 10. Normal distribution in each group was always tested using the Shapiro-Wilk test first for the subsequent choice of a parametric (ANOVA, Student’s t-test) or non-parametric (e.g. Friedman, Mann-Whitney U, Kruskal Wallis or Wilcoxon) test. p-values (α=0.05) were then calculated and multiple testing was corrected for in Prism, as indicated in the figure legends. Values <0.05 were generally considered as statistically significant and denoted by * or # throughout. Comparisons were made to unstimulated control, unless indicated otherwise, denoted by brackets.

**Supplemental Table S1: Primers to detect *MYD88* splice isoforms.**

| **Detection** | **Forward (5' to 3')** | **Reverse (5' to 3')** | **Used concentration** | **Amplicon size** |
| --- | --- | --- | --- | --- |
|  |  |  |  |  |
| Isoform 1/2 | cccagcattgaggaggattgc | ctcaggcatatgccccaggg | 300 nM | 159 bp |
| Isoform 3 | tgggacccagcattgggc | tccttgctctgcaggtaatc | 300 nM | 247 bp |
| Isoform 4 | atgaccccctgggtgcc | gcacctggagagaggctg | 300 nM | 104 bp |
| Isoform 5 | ggacccagcattggtgcc | gcacctggagagaggctg | 300 nM | 109 bp |
| Isoform 8 | agaggttggctagaaggcc | gcacctggagagaggctg | 300 nM | 302 bp |
| GAPDH | agccacatcgctcagacac | gcccaatacgaccaaatcc | 1000 nM | 66 bp |

**Supplemental figure legends**

**Supplemental Figure S1.** (A) Mature RNA and amino acid sequences of MyD88 isoforms 2, 4 and 5 according to reference sequences. Isoforms 4 and 5 show out-of-frame translation compared to the canonical TIR domain sequence due to exon 3 skipping. (B) Mature RNA and amino acid sequences of MyD88 isoforms 1 and 2 showing extra amino acids generated by an alternative donor splice site. (C, D) HEK293T cells were transfected with plasmids for signaling incompetent *MYD88* isoforms 3 (C, n=4) and 5 (D, n=4), followed by stimulation of endogenously expressed TLR5 via flagellin and NF-κB dual luciferase assays performed. A and B are alignments of reference sequences described in Table 1. In C and D one representative of ‘n’ technical replicates is shown. * = p<0.05 according to two-way ANOVA compared to empty vector (EV).

**Supplemental Figure S2.** (A) Primer design to detect isoforms 1-5. (B) Verification of isoform-specific amplification using plasmid constructs. (C) Immunoblot from lymphoma cell lines and primary B cells with short and long exposure (n=2, red GCB, black ABC). (D) Sashimi plot from GCB cells (n=5). (E) RNAseq analysis of DLBCL-clusters: MyD88-like (n=24), BCL2-like (n=9), BCL6-like (n=16) and TP53-like (n=19). Isoform 2 calculated as 1-(sum of all others) and other isoforms are shown as the relative usage from unique splice junctions, see Supplemental Methods. In C one representative of ‘n’ biological or technical replicates is shown and E (Tukey box and whiskers) represent combined data from n biological replicates. * = p<0.05 according to unpaired Student’s t-tests (E).

**Supplemental Figure S3.**

(A) Gating strategy to evaluate B cell purity upon isolation (n=2). (B) Proliferation of stimulated B cells monitored by CFSE (n=2). In A and B one representative of ‘n’ biological replicates is shown.

**Supplemental Figure S4.** (A) Mature RNA and amino acid sequences of MyD88 isoforms 2, 6 and 7 according to reference sequences. Isoforms 6 and 7 show a truncated TIR domain. (B) Mature RNA and amino acid sequences of MyD88 isoforms 2 and 8. Isoform 8 is an hypothetical sequence confirmed by sequencing a BJAB mRNA pool derived PCR amplification product, using primers described in Table S1) Isoform 8 shows an early stop-codon compared to the canonical TIR domain sequence due to intron retention. (C) Representative Sashimi plot from CLL samples (n=289). (D) RT-qPCR analysis of isoforms 8 in stimulated primary B cells (n=5-7). (E) RT-qPCR analysis of isoforms 8 in stimulated primary hMoMacs from the same donors as in C (n=3). A and B are alignments of reference sequences described in Table 1. D and E represent combined data from n biological replicates. * = p<0.05 according to Kruskal Wallis-tests (D) and ordinary one-way ANOVA (E).

**Supplemental Figure S5.** Four representative Sashimi plots from ovarian cancer samples (n = 85). Red shaded boxes point intron retention and orange arcs represents the alternative donor splice site in isoforms 6 and 7.

**Supplementary information references**

Avbelj, M., Wolz, O.O., Fekonja, O., Bencina, M., Repic, M., Mavri, J., Kruger, J., Scharfe, C., Delmiro Garcia, M., Panter, G., Kohlbacher, O., Weber, A.N., and Jerala, R. (2014). Activation of lymphoma-associated MyD88 mutations via allostery-induced TIR-domain oligomerization. *Blood* 124**,** 3896-3904.

Broseus, L., and Ritchie, W. (2020). Challenges in detecting and quantifying intron retention from next generation sequencing data. *Comput Struct Biotechnol J* 18**,** 501-508.

Dobin, A., Davis, C.A., Schlesinger, F., Drenkow, J., Zaleski, C., Jha, S., Batut, P., Chaisson, M., and Gingeras, T.R. (2013). STAR: ultrafast universal RNA-seq aligner. *Bioinformatics* 29**,** 15-21.

Doose, G., Bernhart, S.H., Wagener, R., and Hoffmann, S. (2018). DIEGO: detection of differential alternative splicing using Aitchison's geometry. *Bioinformatics* 34**,** 1066-1068.

Ferreira, P.G., Jares, P., Rico, D., Gomez-Lopez, G., Martinez-Trillos, A., Villamor, N., Ecker, S., Gonzalez-Perez, A., Knowles, D.G., Monlong, J., Johnson, R., Quesada, V., Djebali, S., Papasaikas, P., Lopez-Guerra, M., Colomer, D., Royo, C., Cazorla, M., Pinyol, M., Clot, G., Aymerich, M., Rozman, M., Kulis, M., Tamborero, D., Gouin, A., Blanc, J., Gut, M., Gut, I., Puente, X.S., Pisano, D.G., Martin-Subero, J.I., Lopez-Bigas, N., Lopez-Guillermo, A., Valencia, A., Lopez-Otin, C., Campo, E., and Guigo, R. (2014). Transcriptome characterization by RNA sequencing identifies a major molecular and clinical subdivision in chronic lymphocytic leukemia. *Genome Res* 24**,** 212-226.

Hezaveh, K., Kloetgen, A., Bernhart, S.H., Mahapatra, K.D., Lenze, D., Richter, J., Haake, A., Bergmann, A.K., Brors, B., Burkhardt, B., Claviez, A., Drexler, H.G., Eils, R., Haas, S., Hoffmann, S., Karsch, D., Klapper, W., Kleinheinz, K., Korbel, J., Kretzmer, H., Kreuz, M., Kuppers, R., Lawerenz, C., Leich, E., Loeffler, M., Mantovani-Loeffler, L., Lopez, C., Mchardy, A.C., Moller, P., Rohde, M., Rosenstiel, P., Rosenwald, A., Schilhabel, M., Schlesner, M., Scholz, I., Stadler, P.F., Stilgenbauer, S., Sungalee, S., Szczepanowski, M., Trumper, L., Weniger, M.A., Siebert, R., Borkhardt, A., Hummel, M., Hoell, J.I., and Project, I.M.-S. (2016). Alterations of microRNA and microRNA-regulated messenger RNA expression in germinal center B-cell lymphomas determined by integrative sequencing analysis. *Haematologica* 101**,** 1380-1389.

Hoffmann, S., Otto, C., Doose, G., Tanzer, A., Langenberger, D., Christ, S., Kunz, M., Holdt, L.M., Teupser, D., Hackermuller, J., and Stadler, P.F. (2014). A multi-split mapping algorithm for circular RNA, splicing, trans-splicing and fusion detection. *Genome Biol* 15**,** R34.

Katz, Y., Wang, E.T., Airoldi, E.M., and Burge, C.B. (2010). Analysis and design of RNA sequencing experiments for identifying isoform regulation. *Nat Methods* 7**,** 1009-1015.

Katz, Y., Wang, E.T., Silterra, J., Schwartz, S., Wong, B., Thorvaldsdottir, H., Robinson, J.T., Mesirov, J.P., Airoldi, E.M., and Burge, C.B. (2015). Quantitative visualization of alternative exon expression from RNA-seq data. *Bioinformatics* 31**,** 2400-2402.

Kretzmer, H., Bernhart, S.H., Wang, W., Haake, A., Weniger, M.A., Bergmann, A.K., Betts, M.J., Carrillo-De-Santa-Pau, E., Doose, G., Gutwein, J., Richter, J., Hovestadt, V., Huang, B., Rico, D., Juhling, F., Kolarova, J., Lu, Q., Otto, C., Wagener, R., Arnolds, J., Burkhardt, B., Claviez, A., Drexler, H.G., Eberth, S., Eils, R., Flicek, P., Haas, S., Humme, M., Karsch, D., Kerstens, H.H.D., Klapper, W., Kreuz, M., Lawerenz, C., Lenzek, D., Loeffler, M., Lopez, C., Macleod, R.a.F., Martens, J.H.A., Kulis, M., Martin-Subero, J.I., Moller, P., Nage, I., Picelli, S., Vater, I., Rohde, M., Rosenstiel, P., Rosolowski, M., Russell, R.B., Schilhabel, M., Schlesner, M., Stadler, P.F., Szczepanowski, M., Trumper, L., Stunnenberg, H.G., Kuppers, R., Ammerpohl, O., Lichter, P., Siebert, R., Hoffmann, S., and Radlwimmer, B. (2015). DNA methylome analysis in Burkitt and follicular lymphomas identifies differentially methylated regions linked to somatic mutation and transcriptional control. *Nat Genet* 47**,** 1316-1325.

Lopez, C., Kleinheinz, K., Aukema, S.M., Rohde, M., Bernhart, S.H., Hubschmann, D., Wagener, R., Toprak, U.H., Raimondi, F., Kreuz, M., Waszak, S.M., Huang, Z., Sieverling, L., Paramasivam, N., Seufert, J., Sungalee, S., Russell, R.B., Bausinger, J., Kretzmer, H., Ammerpohl, O., Bergmann, A.K., Binder, H., Borkhardt, A., Brors, B., Claviez, A., Doose, G., Feuerbach, L., Haake, A., Hansmann, M.L., Hoell, J., Hummel, M., Korbel, J.O., Lawerenz, C., Lenze, D., Radlwimmer, B., Richter, J., Rosenstiel, P., Rosenwald, A., Schilhabel, M.B., Stein, H., Stilgenbauer, S., Stadler, P.F., Szczepanowski, M., Weniger, M.A., Zapatka, M., Eils, R., Lichter, P., Loeffler, M., Moller, P., Trumper, L., Klapper, W., Consortium, I.M.-S., Hoffmann, S., Kuppers, R., Burkhardt, B., Schlesner, M., and Siebert, R. (2019). Genomic and transcriptomic changes complement each other in the pathogenesis of sporadic Burkitt lymphoma. *Nat Commun* 10**,** 1459.

Ngo, V.N., Young, R.M., Schmitz, R., Jhavar, S., Xiao, W., Lim, K.H., Kohlhammer, H., Xu, W., Yang, Y., Zhao, H., Shaffer, A.L., Romesser, P., Wright, G., Powell, J., Rosenwald, A., Muller-Hermelink, H.K., Ott, G., Gascoyne, R.D., Connors, J.M., Rimsza, L.M., Campo, E., Jaffe, E.S., Delabie, J., Smeland, E.B., Fisher, R.I., Braziel, R.M., Tubbs, R.R., Cook, J.R., Weisenburger, D.D., Chan, W.C., and Staudt, L.M. (2011). Oncogenically active MYD88 mutations in human lymphoma. *Nature* 470**,** 115-119.

O'donnell, T., Christie, E.L., Ahuja, A., Buros, J., Aksoy, B.A., Bowtell, D.D.L., Snyder, A., and Hammerbacher, J. (2018). Chemotherapy weakly contributes to predicted neoantigen expression in ovarian cancer. *BMC Cancer* 18**,** 87.

Patch, A.M., Christie, E.L., Etemadmoghadam, D., Garsed, D.W., George, J., Fereday, S., Nones, K., Cowin, P., Alsop, K., Bailey, P.J., Kassahn, K.S., Newell, F., Quinn, M.C., Kazakoff, S., Quek, K., Wilhelm-Benartzi, C., Curry, E., Leong, H.S., Hamilton, A., Mileshkin, L., Au-Yeung, G., Kennedy, C., Hung, J., Chiew, Y.E., Harnett, P., Friedlander, M., Quinn, M., Pyman, J., Cordner, S., O'brien, P., Leditschke, J., Young, G., Strachan, K., Waring, P., Azar, W., Mitchell, C., Traficante, N., Hendley, J., Thorne, H., Shackleton, M., Miller, D.K., Arnau, G.M., Tothill, R.W., Holloway, T.P., Semple, T., Harliwong, I., Nourse, C., Nourbakhsh, E., Manning, S., Idrisoglu, S., Bruxner, T.J., Christ, A.N., Poudel, B., Holmes, O., Anderson, M., Leonard, C., Lonie, A., Hall, N., Wood, S., Taylor, D.F., Xu, Q., Fink, J.L., Waddell, N., Drapkin, R., Stronach, E., Gabra, H., Brown, R., Jewell, A., Nagaraj, S.H., Markham, E., Wilson, P.J., Ellul, J., Mcnally, O., Doyle, M.A., Vedururu, R., Stewart, C., Lengyel, E., Pearson, J.V., Defazio, A., Grimmond, S.M., and Bowtell, D.D. (2015). Whole-genome characterization of chemoresistant ovarian cancer. *Nature* 521**,** 489-494.

Puente, X.S., Pinyol, M., Quesada, V., Conde, L., Ordonez, G.R., Villamor, N., Escaramis, G., Jares, P., Bea, S., Gonzalez-Diaz, M., Bassaganyas, L., Baumann, T., Juan, M., Lopez-Guerra, M., Colomer, D., Tubio, J.M.C., Lopez, C., Navarro, A., Tornador, C., Aymerich, M., Rozman, M., Hernandez, J.M., Puente, D.A., Freije, J.M.P., Velasco, G., Gutierrez-Fernandez, A., Costa, D., Carrio, A., Guijarro, S., Enjuanes, A., Hernandez, L., Yague, J., Nicolas, P., Romeo-Casabona, C.M., Himmelbauer, H., Castillo, E., Dohm, J.C., De Sanjose, S., Piris, M.A., De Alava, E., Miguel, J.S., Royo, R., Gelpi, J.L., Torrents, D., Orozco, M., Pisano, D.G., Valencia, A., Guigo, R., Bayes, M., Heath, S., Gut, M., Klatt, P., Marshall, J., Raine, K., Stebbings, L.A., Futreal, P.A., Stratton, M.R., Campbell, P.J., Gut, I., Lopez-Guillermo, A., Estivill, X., Montserrat, E., Lopez-Otin, C., and Campo, E. (2011). Whole-genome sequencing identifies recurrent mutations in chronic lymphocytic leukaemia. *Nature* 475**,** 101-105.

Richter, J., Schlesner, M., Hoffmann, S., Kreuz, M., Leich, E., Burkhardt, B., Rosolowski, M., Ammerpohl, O., Wagener, R., Bernhart, S.H., Lenze, D., Szczepanowski, M., Paulsen, M., Lipinski, S., Russell, R.B., Adam-Klages, S., Apic, G., Claviez, A., Hasenclever, D., Hovestadt, V., Hornig, N., Korbel, J.O., Kube, D., Langenberger, D., Lawerenz, C., Lisfeld, J., Meyer, K., Picelli, S., Pischimarov, J., Radlwimmer, B., Rausch, T., Rohde, M., Schilhabel, M., Scholtysik, R., Spang, R., Trautmann, H., Zenz, T., Borkhardt, A., Drexler, H.G., Moller, P., Macleod, R.A., Pott, C., Schreiber, S., Trumper, L., Loeffler, M., Stadler, P.F., Lichter, P., Eils, R., Kuppers, R., Hummel, M., Klapper, W., Rosenstiel, P., Rosenwald, A., Brors, B., Siebert, R., and Project, I.M.-S. (2012). Recurrent mutation of the ID3 gene in Burkitt lymphoma identified by integrated genome, exome and transcriptome sequencing. *Nat Genet* 44**,** 1316-1320.

Sturm, M., Schroeder, C., and Bauer, P. (2016). SeqPurge: highly-sensitive adapter trimming for paired-end NGS data. *BMC Bioinformatics* 17**,** 208.
