# Supplementary figures and images for: Absence of Non-Canonical, Inhibitory MYD88 Splice Variants in B Cell Lymphomas Correlates With Sustained NF-κB Signaling
Source: Front Immunol. 2021 Jun 7;12:616451. doi: 10.3389/fimmu.2021.616451 (PMC8215704; doi:10.3389/fimmu.2021.616451)

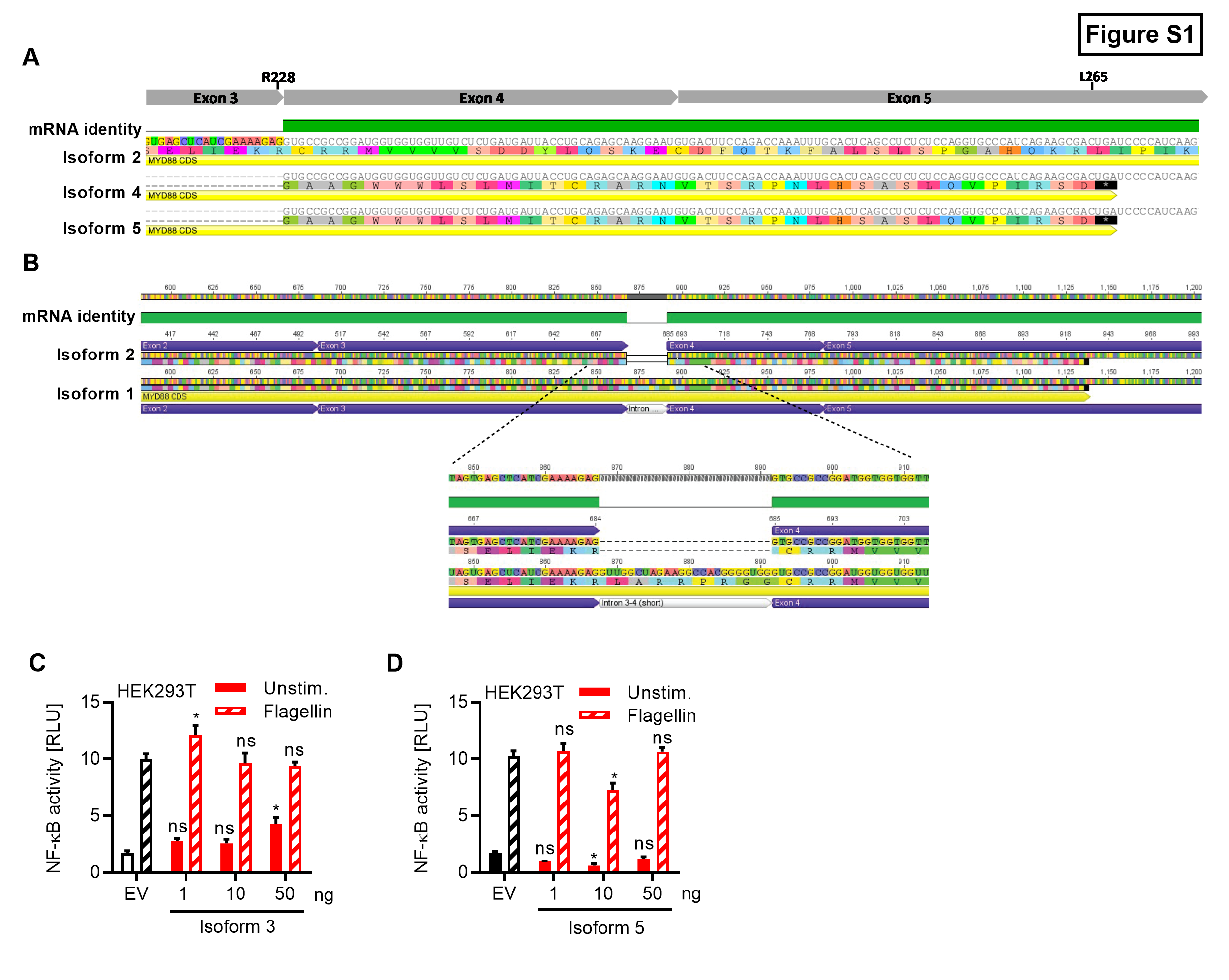

Supplement: Supplementary file 2 [file Image_1.tif]

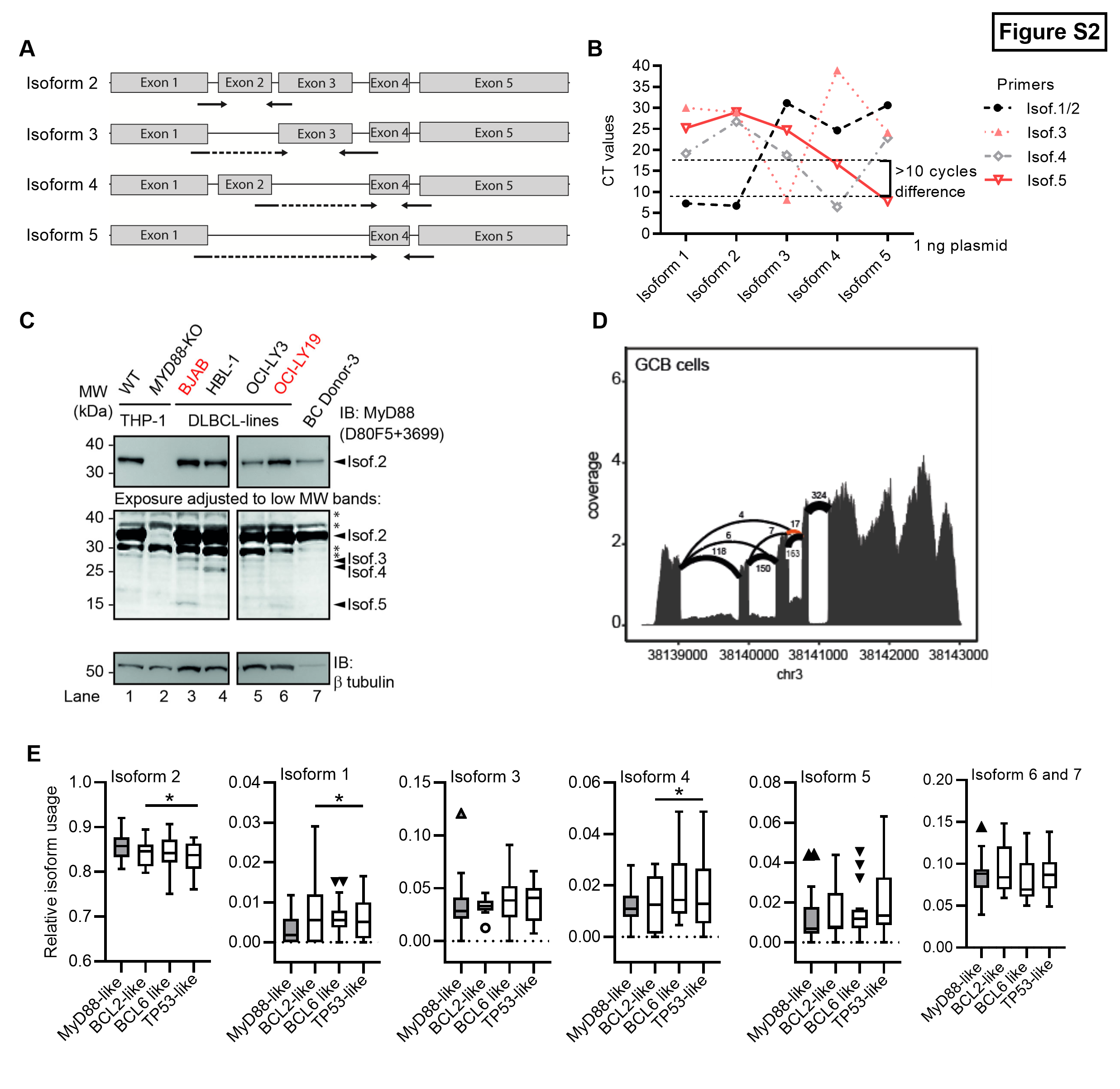

Supplement: Supplementary file 3 [file Image_2.tif]

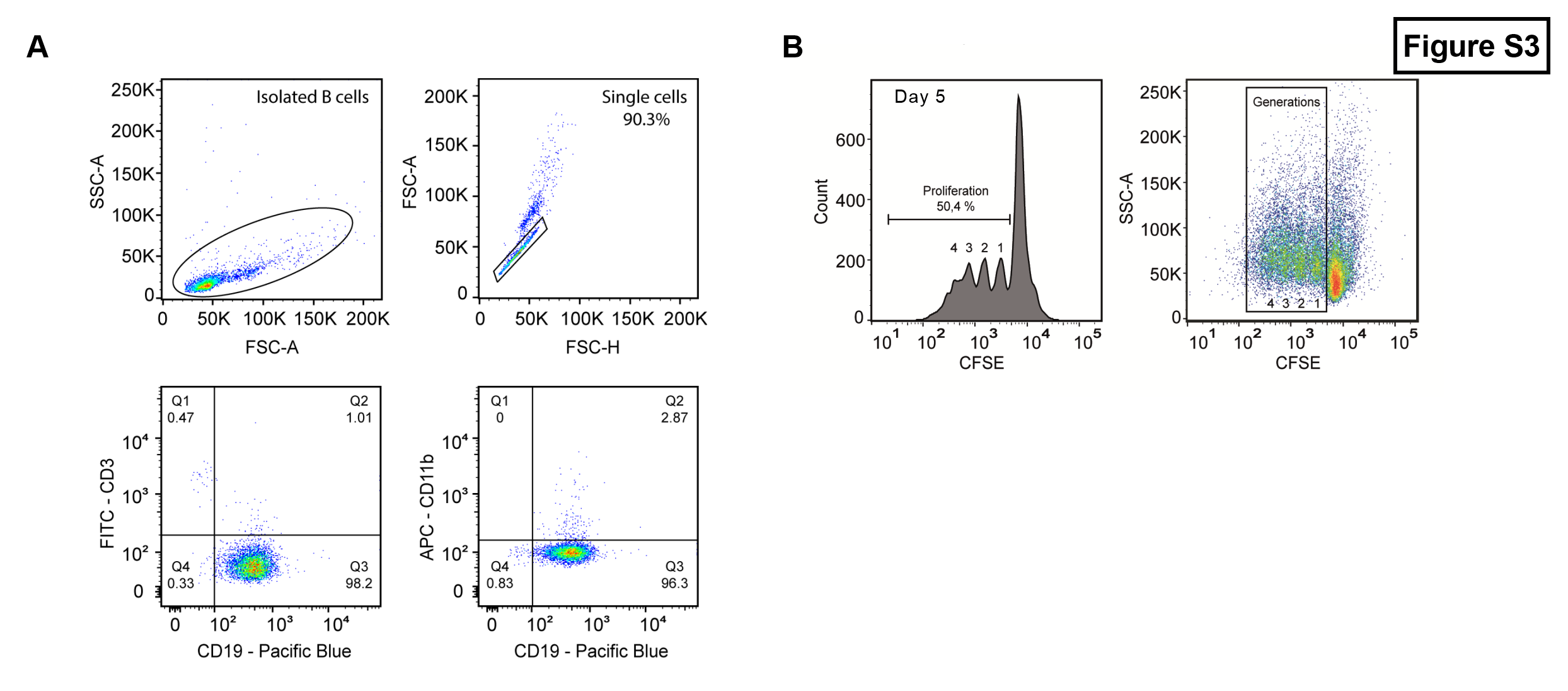

Supplement: Supplementary file 4 [file Image_3.tif]

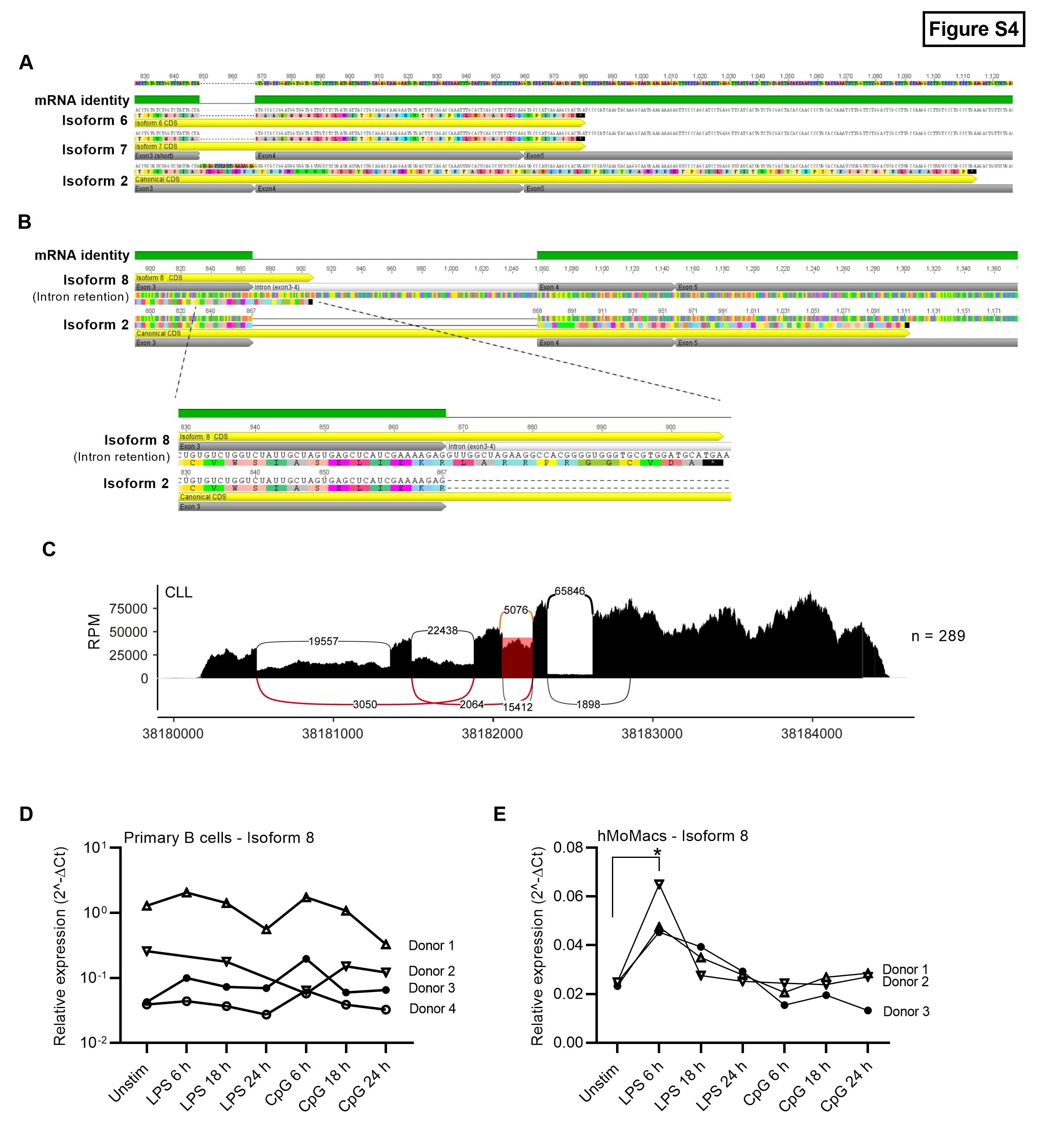

Supplement: Supplementary file 5 [file Image_4.tif]

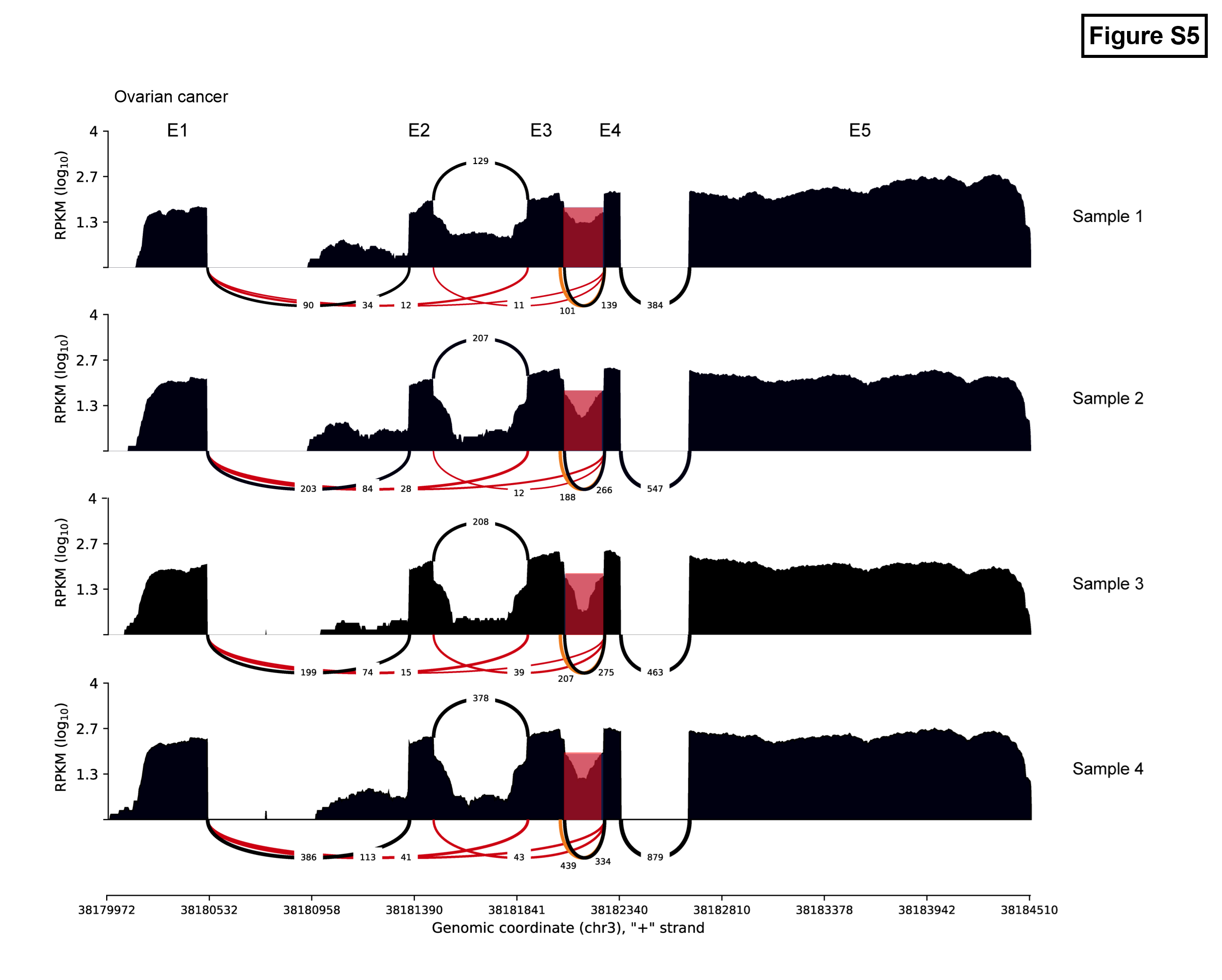

Supplement: Supplementary file 6 [file Image_5.tif]
